# A New Generation of Physicians in Sub-Saharan Africa? Comment on "Non-physician Clinicians in Sub-Saharan Africa and the Evolving Role of Physicians"

**DOI:** 10.15171/ijhpm.2016.97

**Published:** 2016-07-20

**Authors:** Gilles Dussault, Nadia M. Cobb

**Affiliations:** ^1^Global Health and Tropical Medicine, Instituto de Higiene e Medicina Tropical, Universidade Nova de Lisboa, Lisbon, Portugal.; ^2^Office for the Promotion of Global Healthcare Equity, Division of Physician Assistant Studies, Department of Family and Preventive Medicine, University of Utah, Salt Lake City, UT, USA.

**Keywords:** Non-physician Clinicians (NPCs), Medical Education, New Competencies, Sub-Saharan Africa

## Abstract

This commentary follows up on an editorial by Eyal and colleagues in which these authors discuss the implications of the emergence of non-physician clinicians (NPCs) on the health labour market for the education of medical doctors. We generally agree with those authors and we want to stress the importance of clarifying the terminology to describe these practitioners and of defining more formally their scope of practice as prerequisites to identifying the new competencies which physicians need to acquire. We add one new competencies domain, the utilization of new communication technologies, to those listed in the editorial. Finally, we identify policy issues which decision-makers will need to address to make medical education reform work.

In a recent editorial, Eyal et al^1^ advocate a significant reform of medical education in sub-Saharan Africa. They argue that the emergence of healthcare practitioners who produce services traditionally performed by physicians, implies that the latter’s role has to change and that consequently, new competencies must be acquired. These relate to roles and functions such as team management, mentorship, supervision, or planning of services, in addition to more complex clinical ones. They go on to propose that this shift might strengthen the recognition and acceptance of the non-physician clinician (NPC) in health systems through increased inter-professional and team processes. The authors propose that the development of these capacities implies a rethinking of medical education, eg, of the contents of curricula and of learning strategies.

The authors must be commended for stimulating a necessary reflection among educators and policy-makers on how to adapt the education of health professionals in the context of rapid epidemiological and demographic change, of increasing demand for health services and of structural changes in the health workforce. Their contribution is in continuity with the alarm which the Lancet Commission on “Education of health professionals for the 21st century” rang in 2010.^2^

In resource poor regions, including sub-Saharan Africa, not only are the numbers of physicians insufficient to meet the population’s needs, their distribution and migration issues have also led other practitioners to gradually evolve to fill the gap. Scheil-Adlung estimates that 23% of the world’s health workers are in rural areas, while more than 50% of the population lives there.^3^ Numerous studies have shown the NPC to be responsive to rural needs with successful task shifting/sharing.^4-7^ Schneeberger and Mathai note that in Mozambique the assistant medical officers (AMOs) are ‘essential to provide life-saving surgical services in rural areas,’ which in turn decreases unnecessary referrals, decreases delay in care, decreases the workload at referral hospitals, and ultimately costs.^5^ They also note that on average physicians leave their rural postings within 3 years, but 88% of AMO’s stayed more than 7 years.^5^ In this commentary on the discussion by Eyal et al of the impact of the emergence of NPCs in the health labour market on the practice of medicine, we raise questions that need further exploration and discuss the policy implications of reforming medical education.

## The Need for Clearer Definitions and Boundaries

A first issue is the need for clarification of terminology. Eyal et al use NPCs in reference to “practitioners with an education higher than that of nurses, but lower than physicians.” This term was used previously, in a slighter different sense, by Mullan and Frehywot^8^ to describe “providers who are not trained as physicians but who take on many of the diagnostic and clinical functions of medical doctors.” These authors included in this category practitioners who had less skills than physicians but more than basic nurses. Cobb et al^9^ in a recent paper use the term “Accelerated Medically Trained Clinicians” (AMTC) to differentiate cadres trained in the medical model, rather than in the nursing model, that have been included in the NPC terminology previously. In an older paper, Dovlo^10^ referred to “substitute health workers” and to middle-level cadres, a broader notion that covered “cadres who take on some of the functions and roles normally reserved for internationally recognized health professionals such as doctors, pharmacists and nurses.” All authors report important variations in titles corresponding to providers meeting their definition, in training received, tasks performed and level of autonomy. Using an un-differentiated terminology, Dovlo^10^ identified 8 countries in sub-Saharan Africa, and Mullan and Frehywot^8^found some in 25 out of 47 countries surveyed in the same region. Cobb et al^9^ reported the existence of AMTCs in 46 countries globally, in 20 in sub-Saharan Africa, and more than 20 different titles.

We can only agree with Eyal et al when they write **“**All health workers must work together to deliver high quality health services. Major changes to one component of such an integrated system require complementary adaptations elsewhere.” To optimize “the impact of community-based practitioners in the quest for universal health, coverage” Campbell et al^11^ call for a clearer understanding of the ‘front-line’ and community-based health workforce, so as to integrate them fully into health systems, and demonstrate the value of investment in them. Clearly understanding the skills mix of the cadres that have been included in the NPC or mid-level terminology is critical, because otherwise it is difficult to identify precisely which new competencies physicians need to acquire or strengthen. For instance, where NPCs have a high degree of autonomy, physicians will have a different relationship with them and will potentially engage less in mentoring and supervision, that is if they work in an environment where they are part of a team and that support is available in the day-to-day work. In contexts where they work in a more isolated way, like in peripheral or rural areas where in addition to clinical tasks they need to assume management and planning responsibilities, supervision by physicians would still be needed. For example, for the 17 million people of Malawi there are approximately 500 physicians, with 30 in the rural areas. Medical Assistants (primary care focused AMTC) and Clinical Officers (AMTC with increased training in emergency obstetric surgery and surgery) serve in the majority of the country. Henry et al^6^ report that clinical officers and anaesthesia clinical officers “form the backbone of Malawi’s surgical and anaesthetic workforce” with 85%-88% of all surgeries and anaesthesia being performed by these cadres. In other settings where AMTCs (Medical Assistants, Clinical Officer, Community Health Officers, Public Health Officers, etc) run community-based health centres, they often have management responsibilities such as supervising staff, ordering supplies, or general oversight of the functioning of the centre, in addition to seeing patients. Having the support of physicians who have expanded competencies to support this community-based healthcare could greatly increase the efficacy, and potentially quality and safety.

To reform medical education in a manner that prepares future physicians to work in an effective and efficient manner with NPCs, it is important that reformers have a clearer idea of the tasks that each one is expected to perform. While maintaining the cultural specificities of the various cadres of NPCs, some standardization should be promoted, for instance by the World Health Organization (WHO), working with the International Labour Organization (ILO) to ensure that the revision of the *International Standard Classification of Occupations* (ISCO-08) includes these cadres.^12^ A more formal definition and recognition of the scope of practice of NPCs will indeed affect the scope of practice of physicians and of other providers, such as of nurses and possibly of other professionals such as pharmacists. This may take time because such formalization requires the review of existing legislation, which can only be possible if the collaboration of medical associations and councils is obtained. The politics of professional regulation are complex everywhere and sub-Saharan Africa, where NPCs are not always regrouped in associations representing their interests, is no exception.

## Other Examples of New Competencies Needed

This does not mean that the reflection on new competencies domains cannot advance, on the contrary. There is sufficient information on changes taking place in the health sector to identify new learning needs. We suggest to add at least one domain to the preliminary list proposed by Eyal et al: the utilization of new communication technologies, namely ehealth and mhealth. This domain is already included in an implicit manner, but we think it deserves specific attention. NPCs tend to be at the front-line often working alone and unsupported because of their distance from bigger healthcare facilities, as well as of health system structures that fail to define clear paths of communication and supervision. Supporting these clinicians not only increases the quality of care provided, but also serves to motivate them. The possibilities of ehealth and mhealth are abundantly recognized and their utilization is growing rapidly in sub-Saharan Africa.^13^ Future physicians, born in the age of internet, will probably easily integrate the use of new communication technologies in their day-to-day practice; to bring the existing medical workforce to do so may be a more difficult task.

Also, under “leadership and governance,” Eyal et al focus on competencies needed at the level of provider organizations, but others may also be needed at a broader level. For example, physicians need to mobilize and coordinate with other stakeholders in the community to obtain support in vaccination or screening campaigns. In some instances, they may need to interact with aid agencies, which also requires specific skills to negotiate effectively.

## A Challenging Policy Agenda

Beyond the identification of new competencies that physicians need to develop, various policy issues will have to be addressed: how to build the capacity of education institutions to implement reform? Can it be done by the existing staff of educators, knowing that they may also need to develop the new capacities and that, typically, African medical schools rely on part-time staff who may not be able or willing to invest time and energy in mastering new competencies? How physician training can facilitate an understanding of those rural and poor populations which NPCs usually serve, in order to better manage and plan services corresponding to their health needs as well as to adequately supervise NPCs? How communication between the different Ministries responsible for the education of physicians and of other providers, such as NPCs and nurses be improved to ensure a skills-mix aligned on the needs of populations? Is accreditation of programs and institutions the best tool to promote innovation and adaptation to a changing labour market and to the requirements of new delivery models? Should accreditation cover all health professional education to ensure alignment between the education of physicians and of other providers, namely NPCs and nurses? How to bring the existing medical workforce to adopt the new competencies recommended by Eyal et al? What type of continuing professional development activities can be designed and implemented, who will be responsible, how quality will be ensured? Before long another policy issue may emerge, that of the opportunity to create education bridges/ladders for NPCs to become medically qualified.

## Conclusion

There is plenty of evidence that NPCs can provide good quality care to populations with limited access to traditional medical services. These cadres should not be seen as second class substitutes in resource-poor environments. Rather they represent a fit-for-purpose group of providers that add to the traditional skills-mix, being even the “backbone of primary care” in a number of countries or being utilized to increase access to emergency obstetric and surgical care. Investing in NPCs can be a response to health workforce challenges of improved availability, accessibility, acceptability and quality of health workers. By adapting the education of future and existing physicians to new roles, countries would ensure that they, and other providers work to the top of their skills and healthcare systems would be strengthened and more capable of contribution to the Sustainable Development Goal of ensuring healthy lives and promoting well-being for all at all ages. The question, as always, is whether policy- and decision-makers will use the evidence.

## Ethical issues

Not applicable.

## Competing interests

Authors declare that they have no competing interests.

## Authors’ contributions

GD and NMC contributed equally to all stages of the production of this commentary.

## Authors’ affiliations

^1^Global Health and Tropical Medicine, Instituto de Higiene e Medicina Tropical, Universidade Nova de Lisboa, Lisbon, Portugal. ^2^Office for the Promotion of Global Healthcare Equity, Division of Physician Assistant Studies, Department of Family and Preventive Medicine, University of Utah, Salt Lake City, UT, USA.
